# ﻿Description of the female of *Atherimorphalatipennis* Stuckenberg (Diptera, Rhagionidae): the first record of brachyptery in Rhagionidae

**DOI:** 10.3897/zookeys.1178.107357

**Published:** 2023-09-07

**Authors:** John M. Midgley, Burgert S. Muller

**Affiliations:** 1 Department of Natural Sciences, KwaZulu-Natal Museum, 237 Jabu Ndlovu Street, Pietermaritzburg, 3201, South Africa KwaZulu-Natal Museum Pietermaritzburg South Africa; 2 Department of Zoology and Entomology, Rhodes University, P.O. Box 94, Makhanda, 6140, South Africa Rhodes University Makhanda South Africa; 3 Department of Terrestrial Invertebrates, National Museum, Bloemfontein, 9301, South Africa National Museum Bloemfontein South Africa

**Keywords:** Aptery, Lesotho, snipe flies, taxonomy

## Abstract

The genus *Atherimorpha* White, 1915 is a Gondwanan relic, occurring in South America, Southern Africa and Australia. Females are rarely collected, and are not described for more than half of the known species. The female of *Atherimorphalatipennis* Stuckenberg, 1956 was collected for the first time in 2021 and is described here, along with a redescription of the male. We describe the differences from the male, with the reduced wings and poorly defined scutellum the most noteworthy. The female of *A.latipennis* represents the first recorded case of brachyptery in the family Rhagionidae. Possible drivers of brachyptery in Afrotropical Diptera are briefly discussed.

## ﻿Introduction

The genus *Atherimorpha* White, 1915 (Diptera, Rhagionidae) is a Gondwanan relic (Kerr 2010; Kerr and Sinclair 2017) with 51 species recognised (Kerr and Sinclair 2017). Globally, the genus has received sporadic taxonomic attention, with most revisions only addressing regional faunas (Malloch 1932; Paramonov 1962; Nagatomi and Nagatomi 1990), though acknowledging the link between Australia, Africa and South America. Of the 50 extant species, the male is known in 40 cases and the female in 23. Both sexes are only known in 16 species and in three species, it is not clear which sex was described (see Philippi 1865; González et al. 2020). This pattern is also seen in the 12 Afrotropical species, both sexes are known in only four species, and the female is unknown for seven species.

**Figure 1.**
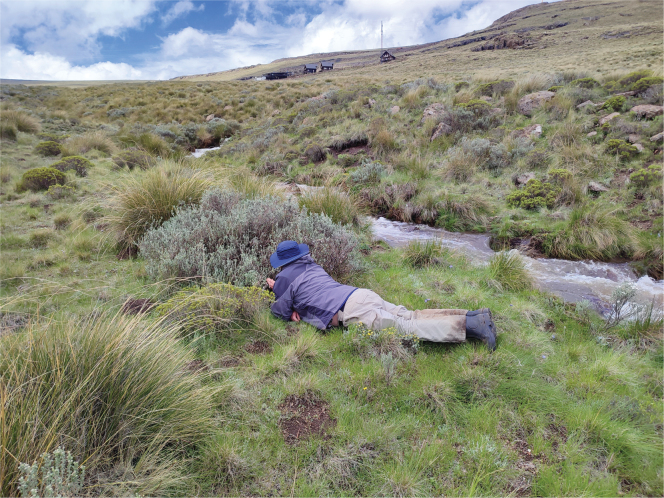
Photograph of site where *A.latipennis* was collected. Afriski Mountain Lodge grounds (28°49.37'S, 28°43.68'E), with first author searching unsuccessfully for additional female specimens. Fifty-one males and single female were collected in 2021 and 68 males in 2022. Photo B. Muller.

Many *Atherimorpha* species are known from mountainous regions, usually near streams (Fig. 1), and emerge as adults for a limited time (Nagatomi and Nagatomi 1990; Kerr 2010). Mountain habitats often drive the evolution of specialised morphology, such as dwarfism in plants (Körner 2003), improved insulation and hypoxia resistance in mammals (Withers et al. 2016), macroptery in birds (Mayr 1963; Moore and Khan 2023) and both brachyptery and macroptery in insects (Leihy and Chown 2020; Moore and Khan 2023). Brachyptery and aptery are driven by both the increased cost of flight and the reduced benefit of flight (Leihy and Chown 2020). Factors increasing the cost of flight include high wind speed, habitat fragmentation, low temperature and low air pressure (Hackman 1964; Leihy and Chown 2020). These factors are all present in the alpine zone of the Lesotho highlands (Partridge et al. 2010; Badger et al. 2015). Predator or competitor release, increased habitat stability, or increased habitat complexity can also drive decreased benefit from flight. These factors can be harder to measure, but are generally not considered to play a role in alpine environments (Leihy and Chown 2020). In insects, the drivers of the evolution of brachyptery and macroptery are the same, with some species evolving to overcome the pressures of these environments (macroptery) while others evolve to avoid them (brachyptery).

We describe the female of *Atherimorphalatipennis* Stuckenberg, 1956 for the first time, redescribe the male to modern standards and provide colour photographs for the first time.

## ﻿Materials and methods

**Figure 2.**
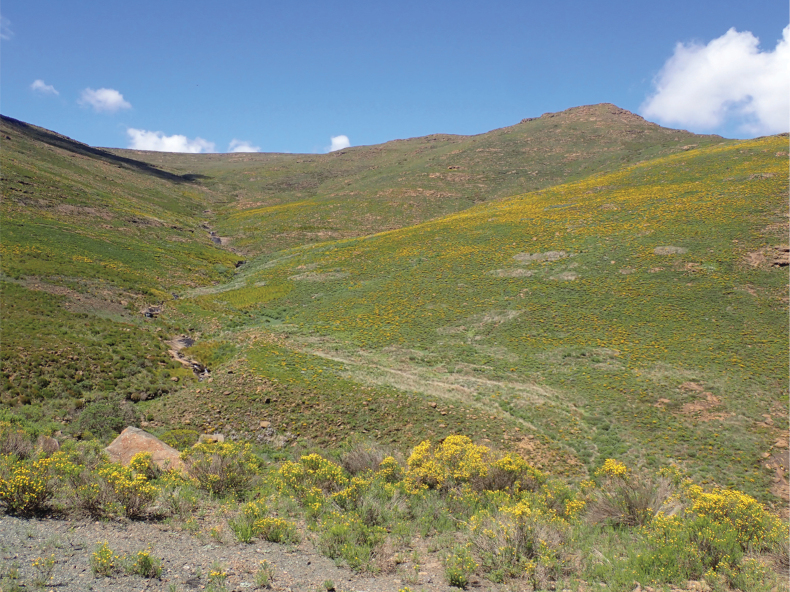
Photograph of site where *A.latipennis* was collected. Tributary of Malibamatso River (28°47.81'S, 28°41.26'E), a single male was collected in November 2022. Photo K. Jordaens, used with permission.

Specimens were collected using sweep nets at Afriski Mountain Lodge and Resort (Fig. 1; 28°49.37'S, 28°43.68'E) in December 2021 and November 2022 and the nearby tributary of the Malibamatso River (Fig. 2; 28°47.81'S, 28°41.26'E) in November 2022. Specimens were also collected at Afriski Mountain Lodge using a Malaise trap in November 2022. The resort falls within the alpine zone at 3032 m a.s.l., and the vegetation is classified as Drakensberg Afroalpine Heathland (Gd10), which is dominated by Fynbos shrubs and grass (Mucina and Rutherford 2006). The Malibamatso tributary is slightly lower at 2872 m a.s.l., but still falls within the Drakensberg Afroalpine Heathland vegetation type.

Specimens were examined using a Leica M80 microscope, photographed using a modified version of the system described by Brecko et al. (2014) and stacked using Helicon Focus 7. Male genitalia were dissected and macerated in lactic acid at 130 °C for 20 minutes, photographed using a Canon 400D and also stacked in Helicon Focus 7. Specimens were identified using the keys and descriptions in Nagatomi and Nagatomi (1990). Terminology follows Cumming and Wood (2017) and Kerr and Sinclair (2017).

Collection codens used in the text are as follows: **BMSA** – National Museum, Bloemfontein, South Africa; **NMSA** – KwaZulu-Natal Museum, Pietermaritzburg, South Africa.

## ﻿Results

### ﻿Identification

All male specimens were identified as *A.latipennis*. The palpi and ocellar tubercle in the freshly collected males match the holotype of *A.latipennis* and not the holotype of *A.longicornu* Nagatomi & Nagatomi, 1990, but comparison of the antennae is not possible as these are missing in the holotype of *A.latipennis* (see Stuckenberg 1956). The antennae and palpi in the female match those of the males collected. The tubercle in the female is less prominent than in the male, but still obviously raised.

### ﻿Taxonomy

#### ﻿ Atherimorpha latipennis

Taxon classificationAnimalia

Stuckenberg, 1956

0066ED9C-FC74-5B22-BAF7-3CA43303623F

Figs 3–4 , 5, 6 , 7, 8 , 9, 10 , 11

Atherimorpha latipennis Stuckenberg, 1956: 144, fig. 1.
Atherimorpha latipennis : Nagatomi and Nagatomi (1990: 64, fig. 59).

##### Material examined.

***Holotype***: Lesotho • 1♂; Thaba-Tseka, nr Sani Pass; L. Bevis leg.; 25 Dec. 1938; NMSA-Dip 053434, NMSA type number 716 (NMSA).

##### Other material.

Lesotho • 20♂♂ 1♀; Butha-Buthe, Afriski Resort, 28°49.37'S, 28°43.683'E; 3–7 Dec. 2021; J. Midgley & B. Muller leg.; sweep net; NMSA-Dip 213161-213181 (NMSA). • 31♂♂; Butha-Buthe, Afriski Resort, 28°49.37'S, 28°43.683'E; 3–7 Dec. 2021; J. Midgley & B. Muller leg.; sweep net; BMSA(D)130376–130406 (BMSA). • 15♂♂; Butha-Buthe, Afriski Resort, 28°49.37'S, 28°43.683'E; 21–24 Nov. 2022; K. Jordaens, J. Midgley, B. Muller & G. Theron leg.; sweep net; NMSA-Dip 217640–217654 (NMSA). • 24♂♂; Butha-Buthe, Afriski Resort, 28°49.37'S, 28°43.683'E; 21–24 Nov. 2022; K. Jordaens, J. Midgley, B. Muller & G. Theron leg.; sweep net; BMSA(D)132356–132379 (BMSA). • 29♂♂; Butha-Buthe, Afriski Resort, 28°49.37'S, 28°43.683'E; 21–24 Nov. 2022; K. Jordaens, J. Midgley, B. Muller & G. Theron leg.; Malaise trap; BMSA(D)132380–132408 (BMSA). •1♂; Butha-Buthe, Afriski: Malibamatso tributary, 28°47.8069'S, 28°41.2561'E; 23 Nov. 2022; K. Jordaens, J. Midgley, B. Muller & G. Theron leg.; NMSA-Dip 217680 (NMSA).

##### Description.

**Figures 3, 4.**
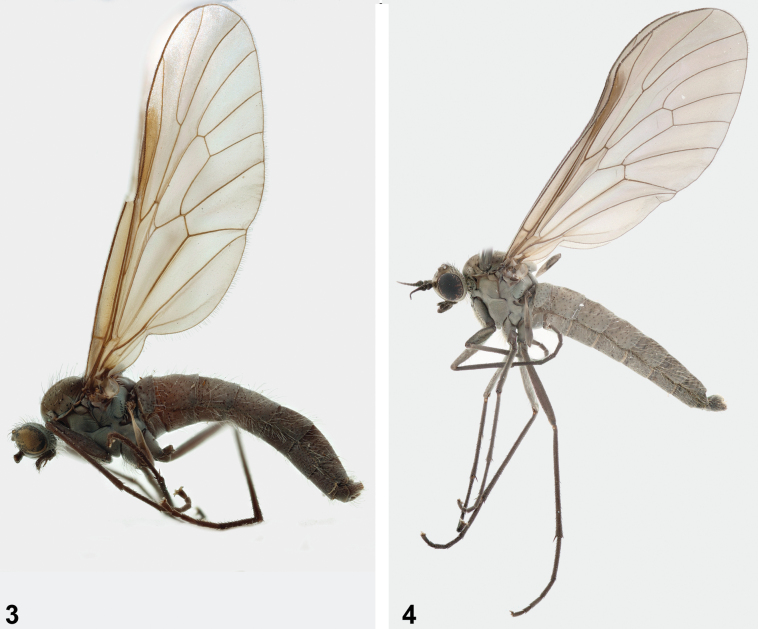
Male of *A.latipennis***3** habitus of holotype (NMSA-Dip 053434) **4** habitus of male collected at Afriski Mountain Lodge (NMSA-Dip 213176).

**Figures 5, 6.**
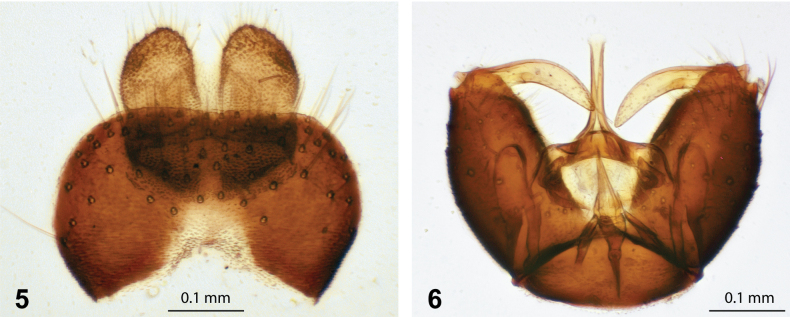
Male of *A.latipennis***5** dorsal view of male epandrium and cerci (BMSA(D)130380) **6** ventral view of male genitalia, with epandrium and cerci removed (BMSA(D)130380).

**Figures 7, 8.**
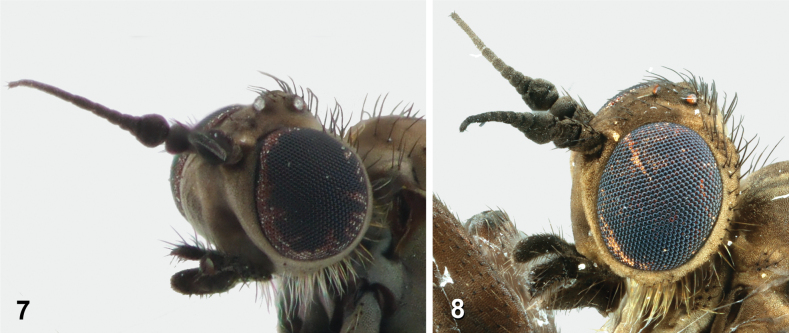
Comparison of the heads of *A.latipennis***7** anterolateral view of male head (NMSA-DIP 213176) **8** anterolateral view of female head (NMSA-Dip 213161)

**Male** (Figs 3–7).

***Length***: Body 3.7–7.4 mm, wing 5.4–7.1 mm.

***Head*** (Figs 3, 4, 7). Overall grey with slight yellowish white pruinosity; frons and ocellar tubercle browner pruinose. Eyes bare; ommatidia similar in size. Ocelli similar size; ocellar tubercle sharply raised, anterior margin almost 90 degrees to frons, as high as diameter of ocelli, posterior margin more gradually sloped, about 45 degrees to frons. Frons with one to three short dark setulae. Upper occiput, vertex and ocellar tubercle with dark setae, lower occiput and gena with pale setulae; proboscis with shorter pale setulae, with some interspersed dark setulae; palpus with dark setulae, longer than width of palpus. Gena narrow. Parafacials separated from clypeus by deep longitudinal sulci; parafacials and clypeus of similar width. Palpus and proboscis darker grey and of similar length. Inner eye margin next to frons with dark mark; lower half of frons with several dark markings. Occiput concave medially. Antennal bases with slight elevated appearance, area surrounding bases with similar colour to parafacials and clypeus; antenna dark brown, almost appearing black; scape and pedicel with short dark setulae dorsally and laterally; flagellomere 1 bare, stylus six-segmented.

***Thorax*** (Figs 3, 4). Scutum greyish brown with three darker brown vittae: middle vitta thin and slightly darker, ending before scutellum, lateral pair ending halfway between suture and scutellum; with scattered long dark setulae; scutum and scutellum clearly separated by suture; scutellum with dark setulae, anterior apical margin with longer pale setulae. Postpronotal lobe grey with whitish pruinosity and dark setulae; pronotum with some scattered pale setulae. Pleura grey with whitish pruinosity, except propleuron and katatergite more yellowish; katatergite with row of pale setulae; propleuron with patch of pale setulae, other pleurites lacking setulae.

***Legs*** (Figs 3, 4). Femorae and tarsi of similar length, tibiae slightly longer. Coxae grey with whitish pruinosity, fore and mid coxae with long pale setulae on anterior surface, hind coxae with setulae on anterior and posterolateral surfaces. Legs overall grey colour, with pale short setulae on most of femorae, some dark setulae toward apex; tibiaе with short and stronger dark setulae; tarsi with darker appearance than rest of leg segments. Pulvilli and empodium of similar size, pulvilli symmetrical.

***Wings*** (Figs 3, 4). Overall light brownish suffused with darker pterostigma in cell *r_1_*; cells *br*, *sc* and base of wing somewhat darker suffused; cell *cua* closed or only very narrowly open; costa with dark setulae along anterior margin of wing, continuing past apex, becoming gradually paler past apex until whitish on anal lobe and alula. *R_1_* with a few dark setulae on dorsal side. Halter with stalk somewhat yellowish-grey and knob darker grey, overall almost as long as fore femur.

***Abdomen*** (Figs 3, 4). Overall grey in colour with slight white pruinosity, covered entirely in only long pale setulae (at least half as long dorsal width of abdomen); half to two thirds height of thorax at join. Terminalia (Figs 6, 7) with gonocoxite grey with orange apex; gonostylus, cercus and parameral sheath orange, hypandrium grey; paired gonocoxites with a somewhat quadrate appearance, bluntly pointed at ventral apex; gonocoxite with inner and outer margins convex, the inner margins less so, almost appearing straight, with strongly developed setae on ventral and apical dorsal surface (with bare ventral basal area); gonostyle cylindrical with apex narrowly tapering, outer margin strongly convex, inner margin gently concave, surface with minute setulae; parameral sheath swollen posterolaterally at base, apically with a trilobate appearance, the lateral lobes obtusely connecting to median lobe; ventral plate broadly ovate, appearing medially divided, particularly at apex; ventral plate covered in minute setulae, except for base; ejaculatory apodeme short and flattened dorso-laterally; lateral ejaculatory process elongated, comparatively longer than ejaculatory apodeme; gonocoxal apodeme elongated; hypandrium with a somewhat semi-circular appearance, apically pointed; hypoproct rather pentagonal, but base with a more rounded appearance; cerci longer than wide, appearing approximately half the length of epandrium.

**Figures 9, 10.**
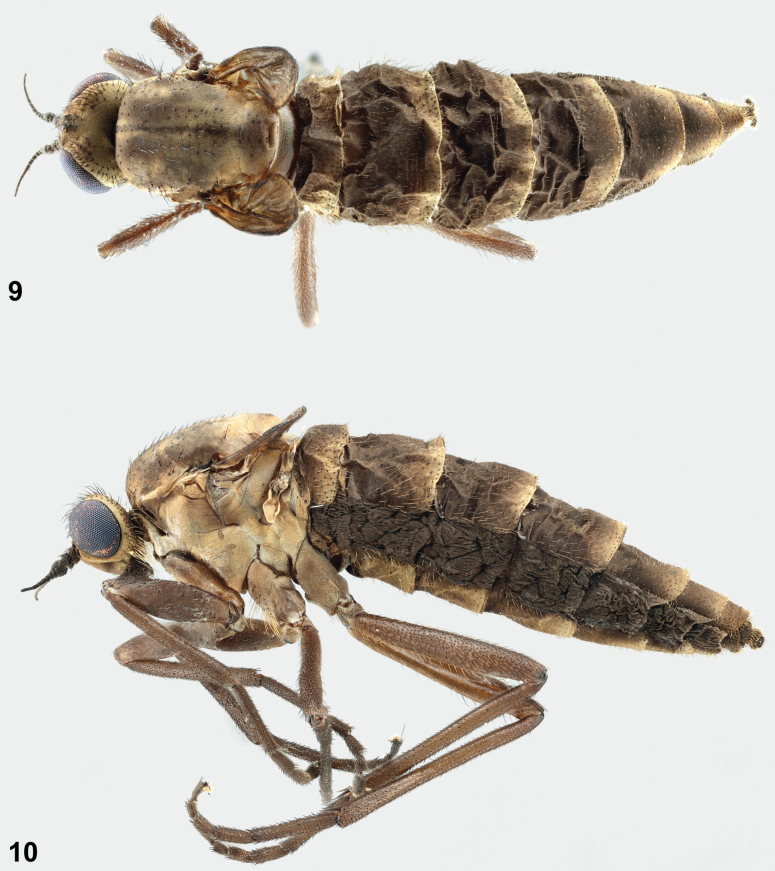
Habitus photographs of female *A.latipennis***9** dorsal view of female specimen (NMSA-Dip 213161) **10** lateral view of female specimen (NMSA-Dip 213161).

**Figure 11.**
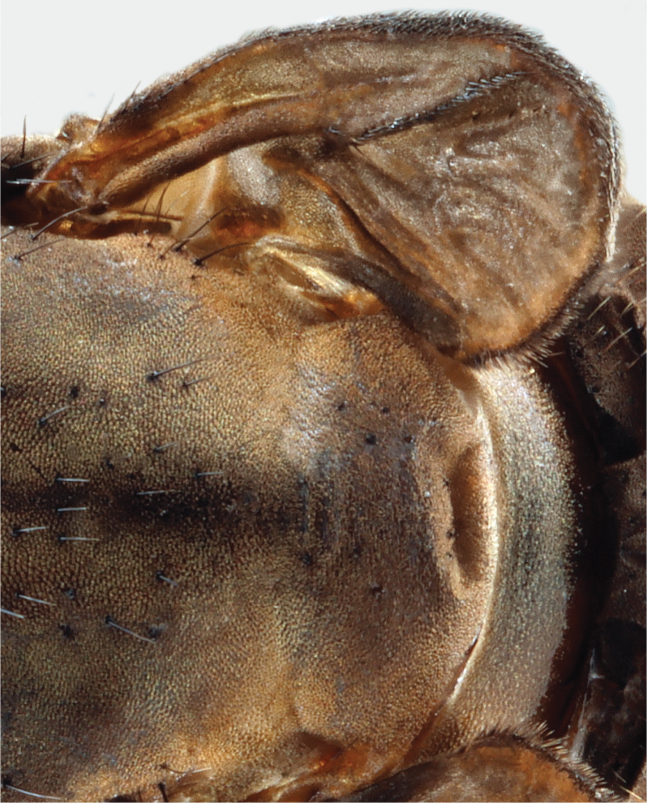
Detail of the wing and scutellum of *A.latipennis* female (NMSA-Dip 213161), showing weakly developed wing veins with setulae and the poorly defined scutellum.

**Female** (Figs 8–11, as for male except as noted below).

***Length***: Body 7.3 mm, wing 1.1 mm.

***Head*** (Figs 8–10).

Overall grey with darker yellow pruinosity than in male. Frons with 13 short dark setulae. Parafacials slightly narrower than clypeus; occiput slightly concave to flat medially.

***Thorax*** (Figs 9–11). Overall, appears overly inflated and stretched; suture between scutum and scutellum indistinct. Scutum greyish brown with three darker brown vittae: middle vitta thin and slightly darker, ending before scutellum; lateral pair less distinct, ending halfway between suture and scutellum. Scutum with scattered short dark setulae; scutellum with a few dark setulae; postpronotal lobe grey with yellowish pruinosity and dark setulae, longer than on disc of scutum. Pleura grey with yellowish pruinosity, except ventral parts of katepisternum and meron whitish. Katatergite with three brown setulae, propleuron with patch of brown setulae, other pleurites lacking setulae.

***Legs*** (Figs 9, 10). Tarsi slightly shorter than femorae; tibiae slightly longer than femorae. Legs overall grey-brown colour.

***Wings*** (Figs 9–11). Greatly reduced, about half length of thorax. Veins indistinct but present; setulae on veins developed, as in male. Halter with stalk and knob somewhat yellowish grey, reduced in size, overall almost as long as width of mid coxa.

***Abdomen*** (Figs 9–11). Overall brown in colour with slight white pruinosity on posterior margins of tergites and sternites, covered entirely in short pale setulae (at most half as long as in male). Abdomen greatly enlarged, about as high as thorax at join. Intersegmental membrane exposed, laterally occupying ¹/3 of height of abdomen. Tergites and sternites weakly sclerotized, appearing wrinkled (possible preservation artefact). Terminalia: cercus, small, grey, rounded; with pale pile about half as long as width of cercus. Not dissected, as only single specimen available.

##### Ecology.

Despite collecting material at several high-altitude sites (see Midgley et al. 2023), specimens of *A.latipennis* were only found in alpine vegetation, suggesting that it is an alpine adapted species.

##### Remarks.

The holotype was published as being at the Durban Natural Science Museum but was donated to the KwaZulu-Natal Museum in the late 20^th^ century. The collection of *Atherimorphalatipennis* at Afriski extends the known range by ~100 km, though both sites fall within the same vegetation type (Drakensberg Afroalpine Heathland). The female will key to brachypterous Hybotidae in the Manual of Afrotropical Diptera adult identification key (Marshall et al. 2017), but can be easily distinguished from it by the enlarged flagellomere 1 and the somewhat narrower, but clearly segmented appearance of flagellomeres 2–7 (Figs 7, 8) (in the form of an arista-like stylus in the Hybotidae), and having two-segmented palpi compared to Hybotidae that are one-segmented. The female keys correctly to *Atherimorpha* in the Rhagionidae chapter (Kerr and Sinclair 2017), though the wing characters in couplet one are indistinct, and to *A.latipennis* using the key in Nagatomi and Nagatomi (1990), though size should not be used to separate this species from *Atherimorphalongicornu* in couplet eight, as we collected specimens of *A.latipennis* smaller than 5.2 mm.

#### ﻿ Atherimorpha longicornu

Taxon classificationAnimalia

Nagatomi & Nagatomi, 1990

49D6759C-FB3C-56DA-AE6C-0F94DAD84C45

Atherimorpha longicornu Nagatomi & Nagatomi, 1990: 64, fig. 59.

##### Material examined.

***Holotype***: South Africa • 1♂; KwaZulu-Natal, Drakensberg Mountains, Royal Natal National Park, 1500 m; B. Stuckenberg and P. Stuckenberg leg.; 14 Nov. 1963; from grassland; NMSA-Dip 052766, NMSA type number 716 (NMSA).

## ﻿Discussion

The brachypterous female of *A.latipennis* is remarkable, being the first recorded case of brachyptery in the family Rhagionidae. Brachyptery has been recorded in 17 families of Diptera in the Afrotropics (Kirk-Spriggs and Sinclair 2017a), and the collection of the female of *A.latipennis* brings this to 18, slightly more than 15% of the families known from the region. Though the percentage of species showing this trait is much lower, the fact that it has evolved in parallel so many times warrants further discussion.

The evolution of brachyptery is often associated with habitat specialisation, though the degree to which this is driven by the increased costs of flight versus the decreased benefit varies between habitats (Hackman 1964; Leihy and Chown 2020). Alpine areas, coastal dunes and polar regions are associated with an increased cost of flight (Darlington 1943), while forests, caves and inquilines or parasitic lifestyles are associated with decreased benefit of flight (Southwood 1962; Roff 1990; Denno et al. 2001). Oceanic islands show attributes of both groups, but the influence of wind (and the associated cost of flight) is the major driver of brachyptery (Leihy and Chown 2020).

In the Afrotropics, both the decreased benefit and increased cost of flight appear to contribute to the evolution of brachyptery. Of the families in which it has been recorded, three include vertebrate inquilines or parasites, three invertebrate inquilines or parasites and six are known from forest habitats (Kirk-Spriggs and Sinclair 2017a, 2017b, 2021). As species of Sphaeroceridae occur in both ant nests and forests, there are 11 families where the lack of benefit is the driver of brachyptery. In contrast, one brachypterous family occurs in coastal dunes, four are recorded from oceanic islands and nine from mountains (now including the Rhagionidae), but as the Chloropidae, Limoniidae and Sphaeroceridae are recorded from both mountains and oceanic islands, 12 families show brachyptery driven by the increased cost of flight (Kirk-Spriggs and Sinclair 2017a, 2017b, 2021; van Zuijlen 2021). It is also worth noting that species of Chloropidae, Empididae, Limoniidae and Sphaeroceridae occur in both groups (Kirk-Spriggs and Sinclair 2017b, 2021).

Hackman (1964) mentioned that brachyptery occurred in one sex in some species but did not suggest possible mechanisms for this. While flight provides insects with evolutionary benefits —e.g., higher dispersal rates, predator avoidance and access to food or reproductive resources — there are undoubtedly costs associated as well —e.g., developmental costs of growing wings, limited use of confined habitats, exposure to severe environmental conditions (Leihy and Chown 2020). Under most conditions, the benefits outweigh the costs, but a shift in this balance drives the loss of flight (Hackman 1964; Leihy and Chown 2020). The primary difference in this regard between males and females is the access to reproductive resources. Increased mating events increases fitness in both male and female insects (Arnqvist and Nilsson 2000), though the increase is not equal in males and females. Females have a finite reproductive capacity and multiple mating events only increases fitness by 30–70% (Arnqvist and Nilsson 2000). Despite the advantage of multiple mating events in females, remaining close to suitable larval habitats is also evolutionarily advantageous. Males on the other hand have a higher theoretical maximum fitness which can only be achieved by locating many females, favouring the evolution of dispersal. Further specimens and studies are needed to confirm this in *A.latipennis*.

The Afrotropical region is large, about 20% of the world’s land surface and ecologically diverse, including eight of the world’s 35 terrestrial biodiversity hotspots (Marchese 2015), and largely underexplored biologically. The alpine zone in southern Africa, particularly Lesotho, has received limited attention to date. Biological surveys of the continent are likely to discover undescribed species, but even the addition of previously undescribed sexes can be remarkable, as shown here. This is particularly true of environments shown to drive atypical morphology. *Atherimorpha* have been collected from mountainous regions in Australia, Southern Africa and South America, yet comparatively few females have been described.

Given that alpine or mountainous environments can result in the evolution of brachyptery and that brachypterous Diptera present a collecting challenge, it is possible that other female *Atherimorpha* are also brachypterous. Future collecting efforts should include multiple techniques, as the usual techniques often rely on passive movement by the target individuals (e.g. Malaise traps) or active searching for flying adults. The inclusion of pitfall trapping, bush beating and other techniques usually used for walking insects may result in the discovery of more brachypterous females.

## Supplementary Material

XML Treatment for Atherimorpha latipennis

XML Treatment for Atherimorpha longicornu

## ﻿Additional information

### Author ORCIDs

John M. Midgley https://orcid.org/0000-0003-1203-3750

Burgert S. Muller https://orcid.org/0000-0002-7304-4050

### Data availability

All of the data that support the findings of this study are available in the main text.
